# TLR9 Mediates Remote Liver Injury following Severe Renal Ischemia Reperfusion

**DOI:** 10.1371/journal.pone.0137511

**Published:** 2015-09-11

**Authors:** Pieter J. Bakker, Angelique M. Scantlebery, Loes M. Butter, Nike Claessen, Gwendoline J. D. Teske, Tom van der Poll, Sandrine Florquin, Jaklien C. Leemans

**Affiliations:** 1 Department of Pathology, Academic Medical Centre, Amsterdam, Netherlands; 2 Center of Experimental & Molecular Medicine, Academic Medical Centre, Amsterdam, Netherlands; 3 Department of Pathology, Radboud University Nijmegen Medical Centre, Nijmegen, Netherlands; National Institutes of Health, UNITED STATES

## Abstract

Ischemia reperfusion injury is a common cause of acute kidney injury and is characterized by tubular damage. Mitochondrial DNA is released upon severe tissue injury and can act as a damage-associated molecular pattern via the innate immune receptor TLR9. Here, we investigated the role of TLR9 in the context of moderate or severe renal ischemia reperfusion injury using wild-type C57BL/6 mice or TLR9KO mice. Moderate renal ischemia induced renal dysfunction but did not decrease animal well-being and was not regulated by TLR9. In contrast, severe renal ischemia decreased animal well-being and survival in wild-type mice after respectively one or five days of reperfusion. TLR9 deficiency improved animal well-being and survival. TLR9 deficiency did not reduce renal inflammation or tubular necrosis. Rather, severe renal ischemia induced hepatic injury as seen by increased plasma ALAT and ASAT levels and focal hepatic necrosis which was prevented by TLR9 deficiency and correlated with reduced circulating mitochondrial DNA levels and plasma LDH. We conclude that TLR9 does not mediate renal dysfunction following either moderate or severe renal ischemia. In contrast, our data indicates that TLR9 is an important mediator of hepatic injury secondary to ischemic acute kidney injury.

## Introduction

Ischemia reperfusion injury (IRI) is a common cause for the development of acute kidney injury (AKI) [1] and is characterized by renal tissue damage and inflammation. Necrosis leads to the release of damage-associated molecular patterns which subsequently activate the innate immune system and trigger an immune response [2].

Toll-like receptors (TLR) are a family of pattern recognition receptors of the innate immune system that are involved in sensing pathogen-associated or damage-associated molecular patterns. In the kidney, TLR2 and TLR4 have been shown to mediate renal inflammation [3–5] and fibrosis [6] following acute renal IRI and chronic obstructive nephropathy. In addition to TLR2 and TLR4, several studies show that TLR9 is expressed in kidney [7,8] and can drive renal pathology in several disease models. Yasadu *et al*. showed that TLR9 deficiency protected against sepsis-induced AKI [9]. In addition, the TLR9 ligand CpG was shown to increase macrophage accumulation and albuminuria in a model for immune complex glomerulonephritis [10] and to worsen renal pathology in a mouse model for lupus nephritis [11].

TLR9 is located in intracellular endosomes of the endoplasmic reticulum and can recognize bacterial and mitochondrial DNA rich in CpG motifs [12–14]. Mitochondrial DNA is released into the circulation upon severe tissue injury and is able to induce systemic inflammation and organ dysfunction through TLR9 [13].

A recent report showed that TLR9 is not involved in renal dysfunction after moderate renal ischemia [15]. However, its role in severe renal ischemia where potentially more and different damage-associated molecular patterns are present remains unclear. Given the expression of TLR9 in the kidney [7,8] and the release of mitochondrial DNA upon severe tissue injury [13], we investigated the role of TLR9 in severe renal ischemia compared to moderate renal ischemia.

We show that TLR9 deficiency resulted in an improved survival of mice and animal well-being in case of severe renal ischemia. These differences did not associate with differences in renal function and tubular necrosis. Improved survival and animal well-being however correlated with reduced plasma mitochondrial DNA content and decreased hepatic injury indicating that TLR9 deficiency reduced remote hepatic injury induced by renal IRI.

## Material & Methods

### Ischemia Reperfusion

Wild-type C57BL/6 male mice age 8–12 weeks were obtained from Charles River. TLR9 deficient (TLR9KO) mice were generated as described previously [16] and subsequently backcrossed for six generations. TLR9 deficiency was confirmed in randomly selected TLR9KO mice (S1 Fig). Renal IR injury was induced as described previously [5]. Briefly, both renal pedicles were clamped for 20 or 30 minutes using microaneurysm clamps through a midline abdominal incision under general anaesthesia (1.25 mg/ml midazolam (Actavis), 0.08 mg/ml fentanyl citrate and 2.5 mg/ml fluanisone (VetaPharma Limited)). After clamp removal, kidneys were inspected for restoration of blood flow. The abdomen was closed in 2 layers and all mice received a subcutaneous injection of 0.1 mg/kg buprenorphin (Temgesic, Schering-Plough). To maintain fluid balance, mice were supplemented with a few drops of sterile 0.9% NaCl intraperitoneal before closing the abdomen. Mice were monitored every twelve hours the first day and daily one day post-IR. Mice were euthanized according to humane endpoints set by the institutional Animal Care Committee (>10% weight loss and immobility). Mice were anesthetized with 2% isofluorane and in case of sacrifice, blood was collected by heart puncture in heparin-coated tubes. No mice died without euthanasia. Euthanized animals were anesthetized followed by cervical dislocation. Kidneys were either snap-frozen in liquid nitrogen or formalin-fixed followed by paraffin embedment. The Animal Care and Use Committee of the University of Amsterdam approved all experiments.

### Animal Discomfort Score

Mice were monitored every 2 hours up to 24 hours after ischemia/reperfusion. Each experimental group was divided into three sub-groups and scores were assigned per sub-group. No heterogeneous behaviour was observed within a sub-group. Each time point, three different scores were assigned per sub-group for appearance, behaviour and reactivity based on literature [17,18] and standardized protocols used by animal caretakers.

The following scores were assigned for appearance; 0: normal, 1: mild pilo-erection, 2: moderate pilo-erection, mild hunched back, 3: severe pilo-erection, hunched back. Behaviour; 0: normal, 1: mildly reduced activity, 2: severely reduced activity, 3: no activity. Reactivity; 0: normal, 1: moves when stimulated, 2: reluctant to move, 3: no movement. An overall animal discomfort score was calculated per sub-group by adding up scores for appearance, behaviour and reactivity. The overall average score per time point per experimental group was calculated by averaging the overall score of the three sub-groups.

### Biochemical analysis

Plasma urea, creatinine, alanine aminotransferase (ALAT), alanine aminotransferase (ASAT) and lactate dehydrogenase (LDH) were measured using standardized clinical diagnostic protocols of the Academic Medical Center Amsterdam.

### Immunohistochemistry

To detect granulocytes, formalin-fixed renal or liver sections were digested with 0,25% pepsin in 0.1 M HCl (Sigma Aldrich) followed by incubation with FITC labelled anti-mouse Ly6G (BD Biosciences), rabbit anti-FITC (Dako) and finally horse radish peroxidase-conjugated goat anti-rabbit IgG. To detect macrophages, formalin-fixed renal sections were boiled in 0.01M pH 6.0 citrate buffer subsequently exposed to a rat IgG2b anti-mouse F4/80 (Serotec). Staining was visualized using a power anti-rabbit poly horse radish peroxidase (Dako) and additionally a rabbit anti-rat antibody (Dako). Ly6G-positive cells were counted in 10 randomly selected non-overlapping high power fields with a 400x magnification. F4/80 staining was quantified using a Dotpro Slidescanner to obtain digital images followed by ImageJ software to quantify the amount of positive pixels.

### Histochemistry

Periodic acid Schiff-diastase (PAS-D) stain was performed as follows. Paraffin-embedded sections were deparaffinized and incubated with 0.25% amylase solution (Sigma Aldrich). Subsequently, slides were incubated with 1% periodic acid (Merck) followed by Schiff reagens (Merck). Counterstaining was carried out using heamatoxyline (Sigma Aldrich). Necrosis was assessed on PAS-D sections by a qualified pathologist using a semi-quantitative score where 0: 0%, 1: 1–10%, 2: 11–25%, 3: 26–50%, 4: 51–75% and 5: more than 75% of tubules are necrotic. Formalin-fixed hepatic sections were deparaffinized, washed in tap water and distilled water and stained in 50% haematoxyline followed by 0.5% eosin for a haemotoxylin&eosin stain.

### DNA electrophoresis

Fifteen sections (20 μm) of a frozen kidney were dissolved in 50 μl of lysis buffer (50 mM Tris-HCl pH 8.0, 20 mM NaCl, 1mM EDTA, 1% SDS, 1 mg/ml proteinase K) and boiled at 55°C for 1.5 hours. Subsequently, 150 μl TE buffer was added and the chromosomal DNA (cDNA) concentrations were measured using a spectrophotometer (Nanodrop). Finally, cDNA was added to the PCR mix (forward and reverse primer, Taq polymerase, MgCl_2_, dNTPs) and the run for 45 consecutive cycles. The end product was visualized on a 1.5% agarose gel with a Proxima 10Phi (Isogen Life Science). The SOCS gene was used as an endogenous reference gene.

### Quantitative PCR

Total RNA was isolated from frozen sections using Trizol (Life Technologies) following the recommended manufacturers’ protocol. Complementary DNA was made by ligation of oligo-dT primers and subsequent polymerization using Taq DNA polymerase (Invitrogen). Finally, cDNA or plasma DNA was quantified in real-time on a Roche LightCycler 480 (Roche Diagnostics) using LightCycler 480 DNA SYBR Green (Roche Diagnostics). To quantify gene expression, cyclophilin G or HPRT was used as an endogenous reference.

### Mitochondrial DNA Measurement

Cell-free plasma was obtained from whole-blood after spinning down for 10 minutes at 10,000 rpm. All DNA present in this plasma was isolated using the QIAiamp Blood Midi Kit following manufacturers’ protocol. Subsequently, genes only present in the mitochondrial DNA were analysed similarly to a quantitative PCR. Primers used were: murine NADH dehydrogenase subunit 1, forward-CAAACCGGGCCCCCTTCGAC-, reverse–CGAATGGGCCGGCTGCGTAT-; murine Cytochrome C oxidase subunit 1, forward–CCAGTGCTAGCCGCAGGCAT-, reverse–TTGGGTCCCCTCCTCCAGCG-. Gene expression of β_2_-microglobulin was used to correct for cellular lysis in plasma.

### Cytokine expression

Snap-frozen kidneys were lysed in a fixed weight/volume ratio using Greenberg Lysis Buffer (75 mM NaCl, 7.5 mM Tris, 0.5 mM MgCl.H_2_O, 0.5 mM CaCl_2_, 0,5% Triton X-100) supplemented with protease inhibitor cocktail (Sigma Aldrich) and subsequently, homogenates were used to determine cytokine levels for KC, MCP-1, TNF-α and IL-1β according to manufacturers’ instructions (R&D Systems).

### Isolation of mitochondrial DNA

Donor wild type male C57Bl/6N mice (Charles River laboratories) were sacrificed under general anaesthesia and whole liver tissue was isolated. Mitochondrial fractions were extracted using cold Isolation Buffer (50mM Sucrose, 200mM Mannitol, 5mM KH_2_PO_4_, 5mM MOPS, 1mM EGTA and 0.1% BSA (all Sigma), pH 7.15) as described before [19]. The obtained mitochondrial fraction was subsequently processed using the QIAamp DNA mini kit (Qiagen) according to the manufacturers’ instructions in order to obtain purified mtDNA and eluted in the appropriate elution buffer. DNA concentrations were determined by spectrophotometer after which samples were stored at -20°C.

### In vitro stimulation of cell lines

All cell lines were cultured in media containing 10% FCS, 2 mM L-glutamine, 100 IU/ml penicillin and 100 μg/ml streptomycin (Invitrogen). In addition, the following media and supplements were used: DMEM/HAM-F12 supplemented with 0.005 mg/ml insulin, 0.005 mg/ml transferrin, 5 ng/ml selenium (Invitrogen) (AML-12), DMEM media (Invitrogen) (RAW 264.7 macrophages). Proximal tubular epithelial cells were isolated from kidneys as described previously [5] and immortalized and primary proximal tubular epithelial cells were cultured using HK2 medium supplemented with 0.005 mg/ml insulin, 0.005 mg/ml transferrin, 5 ng/ml selenium and 1% S1 hormone mixture (Invitrogen). When cells were confluent, they were incubated for 24 hours with either 25 uM CpG-DNA oligonucleotides (Hycult Biotech) or isolated mitochondrial DNA. Supernatants were analysed for KC and TNF-α according to manufacturers’ instructions (R&D Systems).

### Statistics

All data are presented as mean ± standard error of the mean (SEM). Statistical analyses were performed using the non-parametric Mann Whitney test for two group comparisons (in vivo analysis) or the unpaired students t-test (in vitro analysis). Correlation between variables was determined using the non-parametric Spearman test. For all analyses, *p* < 0.05 was considered significant.

## Results

### TLR9 deficiency improves animal well-being and survival after severe renal ischemia

**Fig 1:**
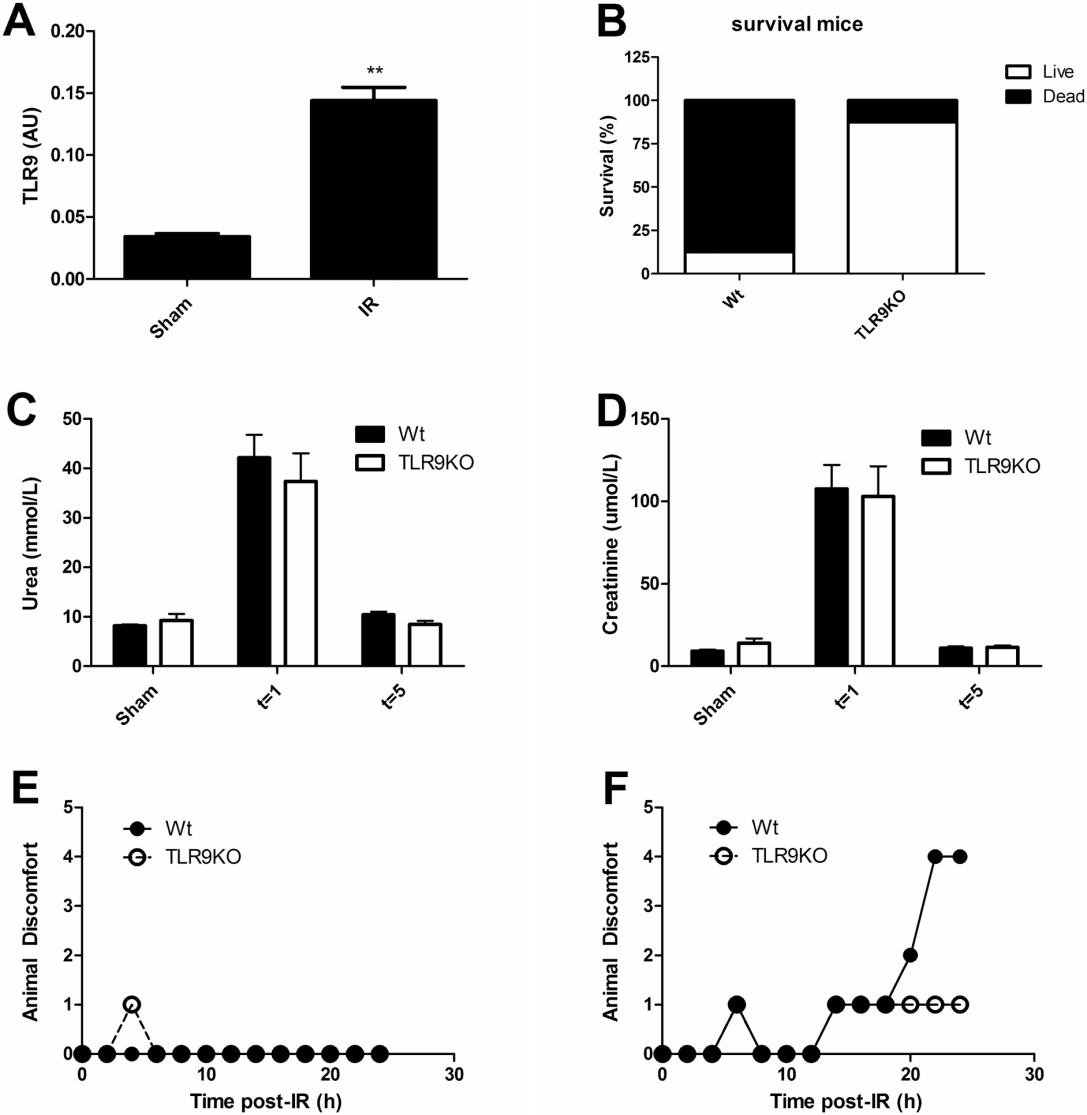
TLR9 deficiency improves survival and animal well being in case of severe renal ischemia. Renal TLR9 gene expression in wild-type mice at five days following sham operation (sham) (n = 4) or moderate ischemia/reperfusion (IR) (n = 9) (A). Survival of wild-type (Wt) and TLR9 deficient (TLR9KO) mice after five days of severe ischemia/reperfusion (B) (n = 8/group). Wild-type and TLR9KO mice were subjected to sham (n = 7/group) or moderate (20 minutes ischemia) renal injury and assessed for renal function after one day (n = 5–7) and five days (n = 8–9), indicated by plasma urea (C) and plasma creatinine (D). Wild-type and TLR9KO mice were subjected to moderate ischemia (20 minutes) (E) or severe ischemia (30 minutes) (F) followed by one day of reperfusion (n = 7-13/group). During reperfusion, mice were monitored the first 24 hours, every two hours with respect to appearance, behaviour, reactivity and anomalies on a scale of 1 to 5. For every time point, the median is shown. AU = arbitrary units. Statistics were performed using Mann-Whitney where p < 0.01 = **.

We observed that TLR9 mRNA expression is induced in the kidney after five days of reperfusion (Fig 1A). Therefore, we induced 30 minutes of renal ischemia followed by five days of reperfusion to study the role of TLR9 on renal pathology. Unexpectedly, 7 out of 8 wild-type mice died on day three to five after reperfusion resulting in 12.5% survival at five days of reperfusion. In contrast, we observed that TLR9 deficiency increased the survival rate after five days of reperfusion (Fig 1B). We reduced the clamping time until all mice survived (20 minutes) however no difference in renal dysfunction after one or five days of reperfusion could be observed between Wt and TLR9KO mice based on plasma urea (Fig 1C) and creatinine (Fig 1D). Therefore, we exposed wild-type and TLR9KO mice to moderate (20 minutes) or severe (30 minutes) ischemia and sacrificed mice at one day of reperfusion to investigate the pathological processes preceding TLR9-mediated death. Following reperfusion, animal discomfort was monitored. This revealed that whereas moderate ischemia did not induce animal discomfort in wild-type and TLR9KO mice (Fig 1E), severe ischemia led to increased animal discomfort only in wild-type but not in TLR9KO mice (Fig 1F). This indicates that TLR9 plays a systemic role following renal ischemia reperfusion which is dependent on the extent of renal ischemia.

### TLR9 deficiency does not reduce renal dysfunction

**Fig 2:**
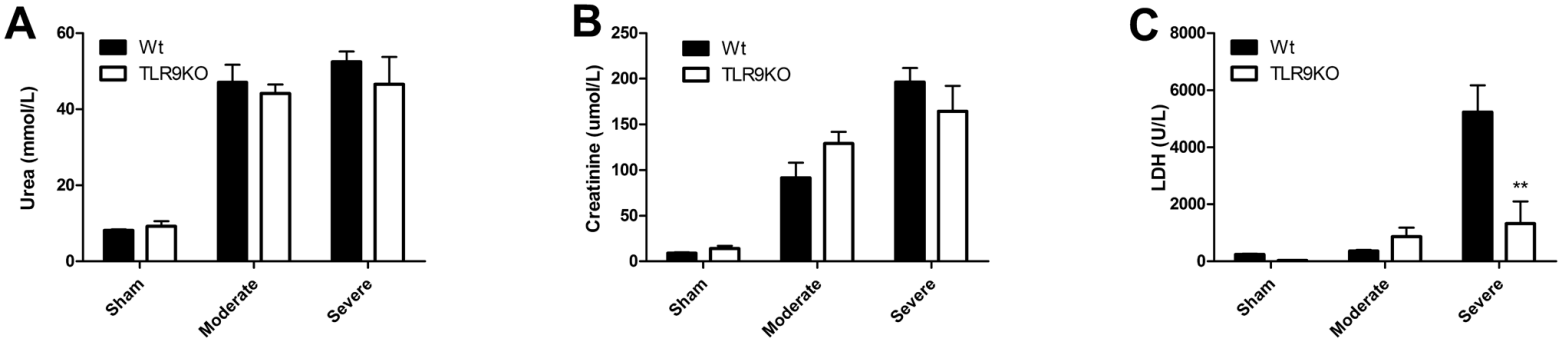
TLR9 deficiency does not reduce renal dysfunction. Wild-type (Wt) and TLR9 deficient (TLR9KO) mice subjected to moderate (20 minutes ischemia) or severe (30 minutes ischemia) renal injury and assessed for renal function after one day, indicated by plasma urea (A) and plasma creatinine (B). In addition, the cellular damage marker lactate dehydrogenase (LDH) was measured in plasma (C) (n = 7-13/group). Statistics were performed using Mann-Whitney where p < 0.01 = **.

We investigated renal function as a potential cause for improved animal well-being and survival. Plasma urea and creatinine levels were increased both after moderate and severe ischemia but did not differ between wild-type and TLR9KO mice (Fig 2A and 2B). Plasma LDH, a general marker of cellular injury, showed a sharp increase in wild-type mice following severe ischemia whereas this was absent in TLR9KO mice subjected to severe ischemia (Fig 2C). These results indicate that plasma LDH levels are dependent on TLR9 and the extent of renal ischemia whereas renal dysfunction is not affected by TLR9 deficiency.

### TLR9 deficiency reduces renal IL-1β levels and granulocyte accumulation

**Fig 3:**
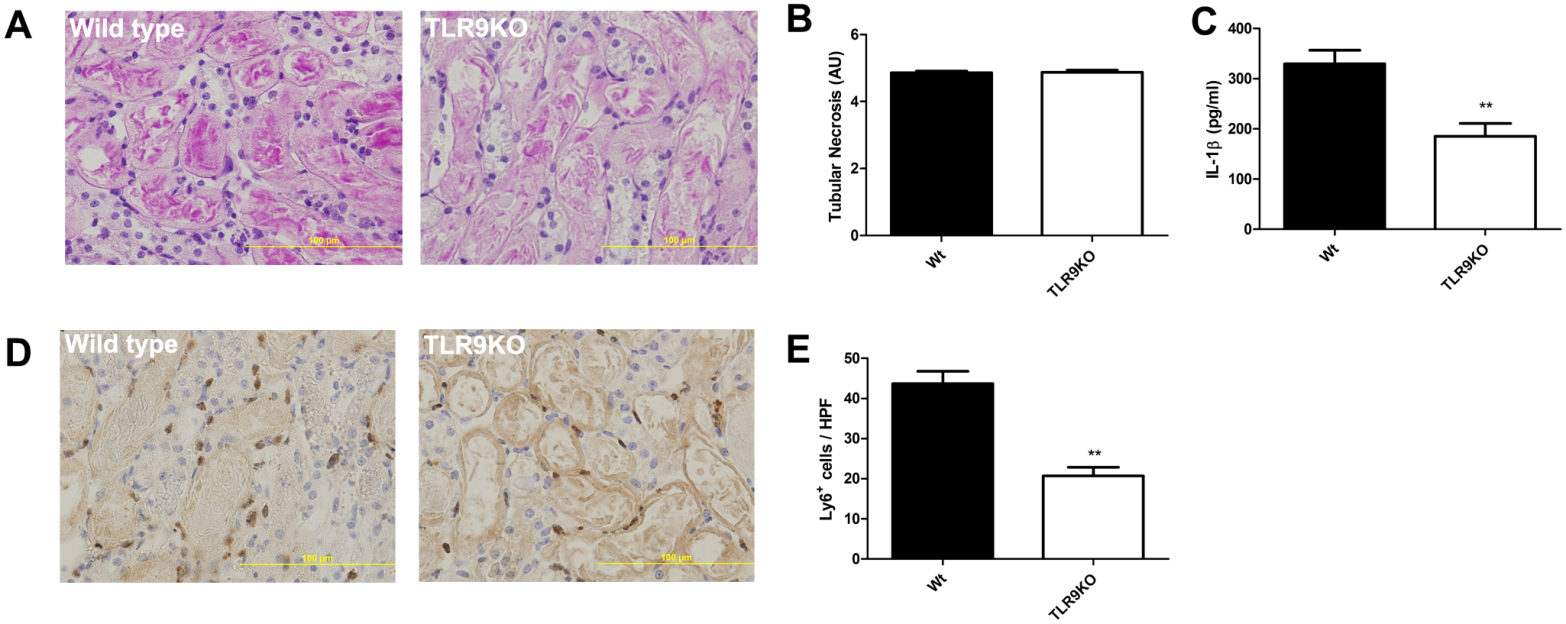
TLR9 deficiency reduces renal IL-1β levels and granulocyte accumulation. PAS-D stained renal sections of wild-type (Wt) and TLR9 deficient (TLR9KO) mice subjected severe ischemia (30 minutes) after one day (A). Semi-quantitative score of tubular necrosis using PAS-D stained renal sections (B). Renal cytokine expression of IL-1β (C) of mice subjected to severe ischemia after one day. Immunohistochemistry using the granulocyte marker Ly6 on sections of mice subjected to severe ischemia after one day (D). Quantification of Ly6-stained sections of mice subjected to severe ischemia after one day (E). For all figures, n = 12-13/group. AU = arbitrary units. Statistics were performed using Mann-Whitney where p < 0.01 = **.

As LDH is released by necrotic tissue, we investigated tubular necrosis as a source of increased plasma LDH levels. Kidneys subjected to severe ischemia showed extensive tubular necrosis which was similar between wild-type and TLR9KO mice (Fig 3A and 3B). We did not observe a difference in renal KC, MCP-1 or TNF-α levels (data not shown). TLR9 deficiency did reduce the amount of renal IL-1β (Fig 3C). Macrophages are able to secrete high amounts of IL-1β [20] and are linked to renal dysfunction [21]. No differences were observed with respect to macrophage accumulation (data not shown) between wild-type and TLR9KO mice in case of severe ischemia. We found that TLR9 deficiency reduced the accumulation of granulocytes in the kidney in case of severe ischemia (Fig 3D and 3E).

### TLR9 deficiency prevents hepatic injury following severe renal ischemia

**Fig 4:**
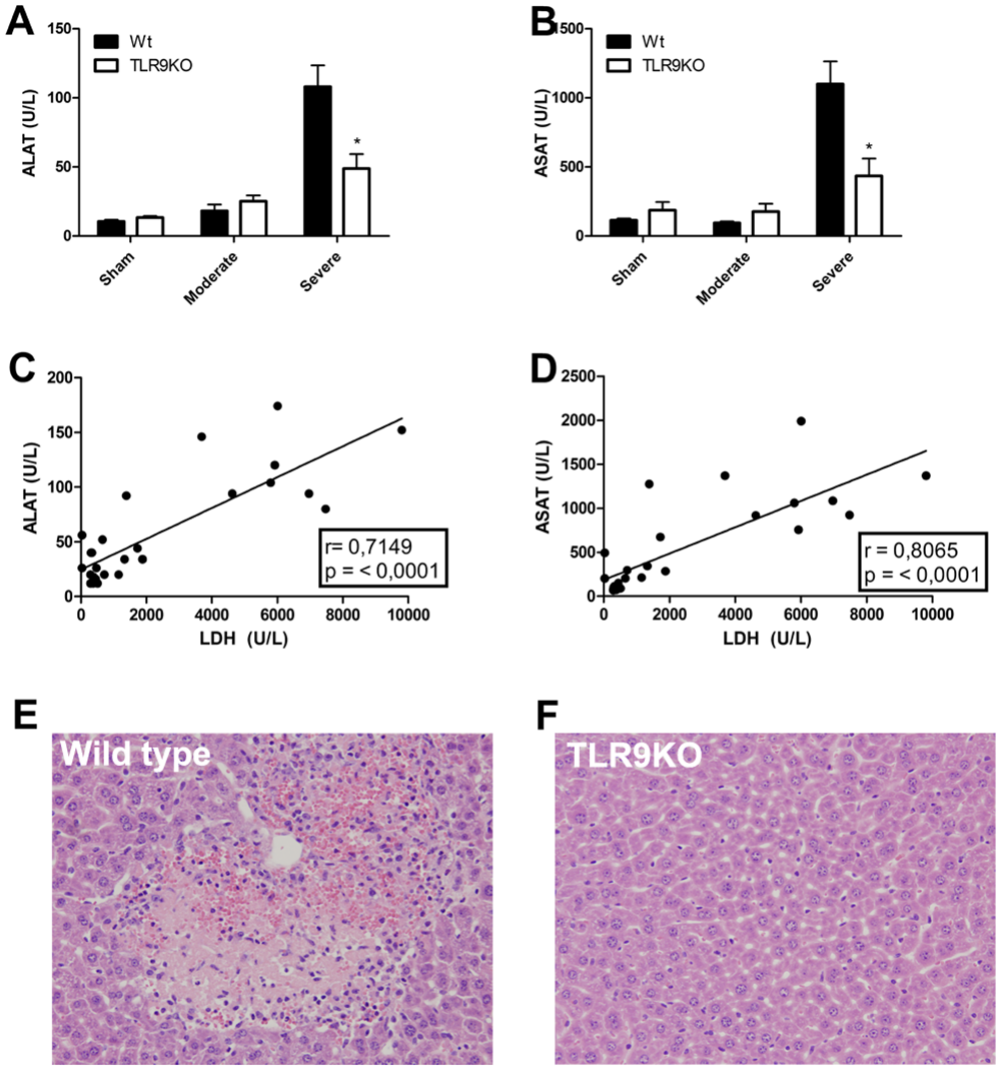
TLR9 deficiency prevents hepatic injury. Liver damage was assessed using plasma alanine aminotransferase (ALAT) (A) and aspartate aminotransferase (ASAT) (B) in wild-type (Wt) and TLR9 deficient (TLR9KO) mice subjected to moderate (20 minutes ischemia) or severe (30 minutes ischemia) renal injury after one day. Plasma LDH levels were correlated to plasma ALAT (C) or plasma ASAT (D) levels (n = 7-13/group). Haematoxylin-eosin staining of liver sections (200x) of wild-type (E) and TLR9KO (F) mice subjected to severe renal ischemia after one day. Correlations were performed using the non-parametric Spearman test. Two group comparisons were performed using Mann-Whitney where p < 0.05 = *.

AKI associates with high morbidity and mortality, in part because of extra-renal complications such as liver dysfunction [22,23]. Therefore, we assessed plasma ALAT and ASAT to assess liver damage. We observed that both plasma ALAT (Fig 4A) and ASAT (Fig 4B) increased in wild-type mice following severe renal ischemia demonstrating that renal IRI can cause remote hepatic injury. Interestingly, we observed that TLR9 deficiency prevents hepatic injury as seen by decreased plasma ALAT and ASAT levels in case of severe renal ischemia. Plasma ALAT and ASAT levels in wild-type and TLR9KO mice correlated highly significantly with plasma LDH levels (Fig 4C and 4D). Furthermore, we assessed liver histology and found that wild-type mice showed necrotic foci (Fig 4E) which corresponded to 1–4% of the total liver area whereas TLR9KO did not show necrosis (Fig 4F). Immunohistochemistry revealed a marginal neutrophil influx in Wt and TLR9KO mice (0.37 ± 0.09 and 2.24 ± 0.62 granulocytes per high power field) (S2 Fig). This indicates that increased plasma LDH levels are related to hepatic injury, and that renal IR-induced remote hepatic injury is affected by TLR9.

### Severe renal ischemia increases plasma mitochondrial DNA content in a TLR9-dependent fashion

**Fig 5:**
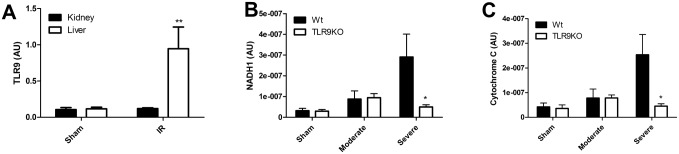
Renal ischemia reperfusion leads to TLR9-dependent release of mitochondrial DNA. Renal and hepatic TLR9 expression in sham-operated mice and in mice after one day of ischemia/reperfusion (IR) (A) (n = 4-9/group). Expression of the mitochondrial genes NADH subunit 1 (NADH1) (B) and mitochondrial Cytochrome C (C) as markers for circulating mitochondrial DNA in wild-type (Wt) or TLR9 deficient (TLR9KO) mice subjected to moderate (20 minutes) or severe (30 minutes) ischemia after one day (n = 7-13/group). AU = arbitrary units. Two group comparisons were performed using Mann-Whitney where p < 0.05 = *, p < 0.01 = **.

Since TLR9 deficiency reduced remote hepatic injury following severe renal ischemia, we finally focused on a soluble mediator which may connect renal ischemic injury with remote hepatic injury. TLR9 is able to sense mitochondrial DNA which is released following severe tissue injury [13]. We observed that TLR9 expression is induced in the liver but not in the kidney one day post-IR (Fig 5A). Proximal tubular epithelium expresses TLR9 to a similar extent as whole kidney whereas hepatocytes have low TLR9 expression compared to whole liver. In contrast, macrophages express very high levels of TLR9 (S3 Fig). Therefore, we speculated that mitochondrial DNA mediates remote hepatic injury through hepatic TLR9 after severe renal ischemia. Using a method to detect mitochondrial DNA in the circulation as described previously [13], we observed that DNA isolated from plasma contained mitochondrial DNA as shown by the expression of the mitochondrial genes NADH subunit 1 (Fig 5B) and cytochrome C (Fig 5C) and showed a tendency towards increased levels in case of moderate ischemia. Interestingly, a sharp increase in mitochondrial NADH1 and Cytochrome C was observed in case of severe ischemia in wild-type but not in TLR9KO mice. Gene expression of β_2_-microglobulin was used to assess cellular lysis but similar between all groups (data not shown).

### TLR9 ligands CpG and mitochondrial DNA activate hepatocytes and macrophages

**Fig 6:**
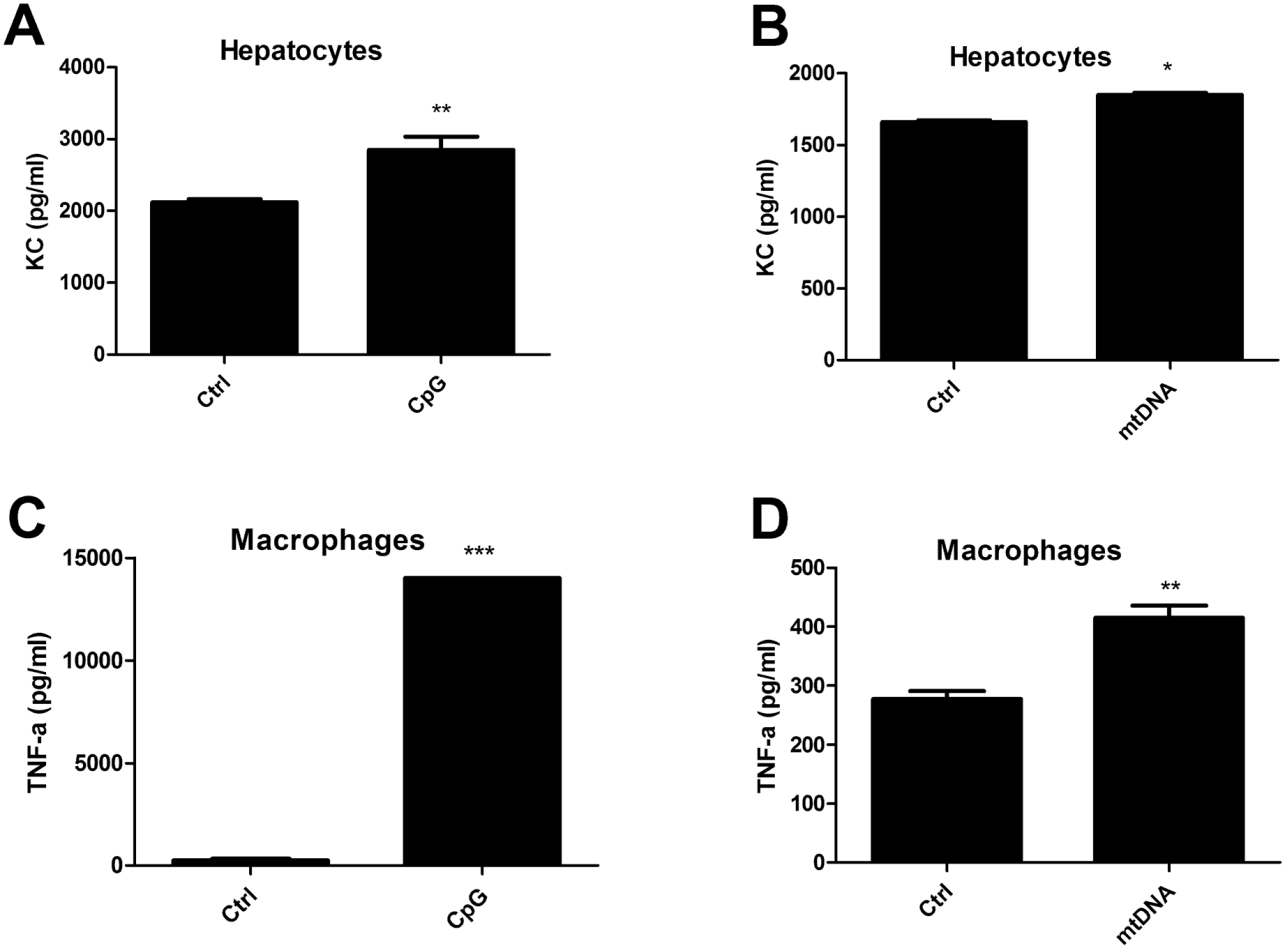
CpG and mitochondrial DNA activate hepatocytes and macrophages. The hepatocyte cell line AML12 (A,B) (n = 3) and macrophage cell line RAW 264.7 (C,D) (n = 3) were stimulated with 25 uM CpG-oligonucleotides (CpG) (A,C) or 50 ng/ul mitochondrial DNA (mtDNA) (B,D) for 24 hours and subsequently KC or TNF-α was measured in the supernatants of respectively AML12 hepatocytes or RAW 264.7 macrophages. Two group comparisons were performed using unpaired students t-test where p < 0.05 = *, p < 0.01 = ** and p < 0.001 = ***.

To elucidate which cell types could respond to mitochondrial DNA, we performed an in vitro stimulation with CpG oligonucleotides or isolated mitochondrial DNA. We observed that CpG (Fig 6A) and mitochondrial DNA (Fig 6B) are able to activate hepatocytes and release the chemokine KC compared to unstimulated cells. Furthermore, we observed that both CpG (Fig 6C) and isolated mitochondrial DNA (Fig 6D) are able to activate macrophages and significantly induce the release of the pro-inflammatory cytokine TNF-α compared to unstimulated macrophages. Proximal tubular epithelial cells did not secrete KC nor TNF- α in response to either CpG or mitochondrial DNA (data not shown).

## Discussion

Here, we show that TLR9 deficiency improved animal well-being and survival following severe renal ischemia. Renal dysfunction, inflammation and tubular necrosis was not dependent on TLR9 suggesting an extra-renal cause. We show that renal IR induced hepatic injury only following severe ischemia which correlated with increased plasma LDH levels and hepatic necrosis. Furthermore, we found that TLR9 deficiency reduced remote hepatic injury. Severe renal ischemia increased plasma mitochondrial DNA levels and this increase was dependent on TLR9. Together, our results indicate that circulating mitochondrial DNA and TLR9 are detrimental mediators between injured kidney and remote hepatic injury. We therefore propose that TLR9-mitochondrial DNA interaction is a potential mechanism of kidney-liver crosstalk during ischemic AKI.

The role of TLRs in renal disease is complex. Although TLR9 deficiency protects against sepsis-induced AKI [9], no role for TLR9 could be established in a model of renal IRI [15] or moderate renal IRI (this study). We showed that TLR9 expression was increased after five days of reperfusion. TLR9 gene expression is linked to protein localisation in the kidney [7]. In line with gene expression data, we observed that in mice subjected to severe renal ischemia, TLR9 deficiency improved survival after five days of reperfusion. Furthermore, TLR9 deficiency improved animal well-being after one day of reperfusion only in case of severe renal ischemia and not in case of moderate renal ischemia. This indicates that the detrimental role of TLR9 is dependent on the extent of renal injury. This observation is in line with the paper of Li *et al*. where TLR9 deficiency did not reduce renal dysfunction in a model of moderate renal ischemia based on plasma creatinine levels [15]. In contrast to TLR9, TLR2 and TLR4 were shown to play a detrimental role following renal IRI [3–5] indicating that the pathological mechanism of renal IR injury is mediated by specific TLR family members.

Renal KC, MCP-1 or TNF-α levels were not changed in TLR9KO mice compared to wild-type mice after severe renal IRI which was in line with previous observations [15] and coincided with similar renal function. In contrast, TLR9 deficiency reduced renal IL-1β levels. Macrophages are potent induces of IL-1β [20] however macrophage influx was similar between wild-type and TLR9KO mice. Reduced renal IL-1β levels were in concordance with a previous study where TLR9 deficiency reduced the expression of IL-1β in a model of acetaminophen-induced liver injury [24].

Although renal KC and tubular necrosis were similar in both mice strains, granulocyte influx in kidneys was reduced in TLR9KO mice. A report published by Itagaki et al. shows that bacterial DNA which is rich in CpG motifs and a TLR9 ligand, is able to change endothelial permeability and facilitate neutrophil extravasation [25]. A similar phenomenon might take place via mitochondrial DNA which is also rich in CpG motifs, released upon severe tissue injury and known to activate TLR9 [13]. Indeed, we observed that in case of severe ischemia, TLR9KO mice have less renal granulocytes and less circulating mitochondrial DNA suggesting granulocyte influx is rather a reflection of circulating mitochondrial DNA instead of KC expression or renal injury.

Renal function was to a similar extent impaired in wild-type and TLR9KO mice when subjected to moderate or severe ischemia and was therefore not responsible for differences in animal well-being and survival. Instead, differences in plasma LDH levels after severe renal IR indicated that cellular necrosis could underlie this phenotypic difference. Since tubular necrosis was similar in both mice strains, we subsequently investigated extra-renal sources of circulating LDH. AKI is despite renal replacement therapy still an important cause for morbidity largely due to extra-renal complications. As AKI has an effect on liver function [23,26,27] and hepatic IRI is dependent on TLR9 [28], we next analysed circulating hepatic injury parameters and hepatic necrosis. We found that TLR9 deficiency prevented hepatic injury as seen by reduced plasma ALAT and ASAT and reduced hepatic necrosis in case of severe renal injury. Moderate renal ischemia did not induce hepatic injury indicating a delicate balance between renal ischemia and hepatic injury. Our data is in concordance with previous data where renal IR leads to hepatic injury as indicated by increased plasma LDH, ALAT and ASAT levels [29]. We extend this paradigm by including TLR9 as a mediator of hepatic injury secondary to ischemic AKI. Similarly, it was shown that TLR9 functioned as a bridge between the release of damage-associated molecular patterns from the intestine and hepatic dysfunction [30]. This suggests that TLR9 is a crucial mediator of hepatic injury independent of the origin of damage-associated molecular patterns. Other sources of plasma LDH than liver cannot be excluded.

Mitochondrial DNA can activate TLR9 [14] and is released upon severe tissue injury [13]. Moderate renal ischemia showed a trend towards increased circulating mitochondrial DNA whereas severe renal ischemia induced high levels of circulating mitochondrial DNA. TLR9 deficiency prevented an increase in hepatic injury, circulating LDH and mitochondrial DNA content secondary to AKI. The increased hepatic expression of TLR9 is in line with the observation that TLR9 deficiency alleviates hepatic dysfunction but not renal dysfunction where there is no increased TLR9 expression after one day of reperfusion. Granulocytes were virtually absent in damaged livers of Wt mice therefore hepatocytes and resident macrophages are of further interest. In vitro data supports a role for mitochondrial DNA upstream of TLR9 activation through the induction of chemokines by hepatocytes and TNF-α by resident macrophages. TNF-α is a well-known inducer of hepatic injury (32). However, this could not be definitely demonstrated in vivo. Whether mitochondrial DNA release is also the result of TLR9 activation remains unknown. Our data point towards a role of mitochondrial DNA upstream of TLR9 activation in hepatocytes and Kupfer cells without excluding the possibility that TLR9 activation also leads to mitochondrial DNA release. We speculate that in case of moderate renal ischemia, mitochondrial DNA is released by the kidney at levels that are not sufficient to cause hepatic injury. In case of severe renal ischemia, mitochondrial DNA is released in high amounts in the circulation by the kidney and causes TLR9-dependent hepatic injury. Subsequently, hepatic injury will lead to increased levels of circulating mitochondrial DNA creating a vicious inflammatory cycle.

Hepatocytes are able to respond to the TLR9 ligands CpG and mitochondrial DNA where proximal tubular epithelial cells are not despite increased TLR9 expression. TLR9 activation is regulated through receptor compartmentalization [31]. Here, carrier proteins can differentiate ‘self’ from ‘non self’ and direct ‘non self’ nucleic acids towards TLR9. Furthermore, the full-length TLR9 protein is cleaved by proteases in the endolysosome to form the functional truncated TLR9 protein indicating that merely TLR9 gene expression is not sufficient to have a functional receptor [32]. Therefore, the local presence of carrier proteins and tissue-specific post-translational processing can induce tissue-specific differences in TLR9 responsiveness and might play a role in liver- versus kidney-specific TLR9 responsiveness. Circulating mitochondrial DNA might be an interesting link between TLR9 following renal IR and remote hepatic injury however other ligands may be involved. It was shown that circulating levels of histones are increased following hepatic IR and neutralization of these histones alleviated hepatic injury [33]. It was concluded that histones similar to HMGB1 [34], can bind DNA which subsequently activates TLR9. This observation supports the detrimental role for DNA in TLR9 activation in the context of renal IR but also suggests that a scaffold protein is necessary to direct a TLR9 ligand to the receptor. An additional mechanism capable of activating TLR9 is via autoantibody production and the formation of immune complexes. It was also shown that TLR9 controls autoantibody formation [35] and that bacterial DNA worsens immune complex glomerulonephritis [10]. Antibodies could therefore also play a role here. More research is needed to investigate other mechanisms that could lead to TLR9 activation.

Here, we show that TLR9 deficiency improves animal well-being and survival following severe renal ischemia. This could not be attributed to an effect of TLR9 deficiency on renal function, inflammation or tubular necrosis. Rather, TLR9 deficiency prevented renal ischemia-induced hepatic injury which correlated with reduced circulating LDH and mitochondrial DNA levels. Together, we speculate that the TLR9-mitochondrial DNA axis is an important mediator of liver injury secondary to ischemic AKI.

## Supporting Information

S1 Fig TLR9 deficiency of wild-type and TLR9KO mice. We randomly selected wild-type (Wt) and TLR9-deficient mice (TLR9KO) and analysed genomic DNA for the presence of the deletion in TLR9. SOCS was used as an endogenous reference.
(TIF)

S2 Fig Granulocyte accumulation in mice subjected to severe AKI. Liver sections of wild-type (Wt) and TLR9 deficient (TLR9KO) mice subjected to severe (30 minutes) ischemia and one day of reperfusion were analysed for Ly6G+ granulocyte accumulation at 400x magnification (high power field).
(TIF)

S3 Fig Tissue-specific TLR9 expression in liver and kidney. TLR9 gene expression was measured in whole kidney, whole liver, primary proximal tubulus epithelial cells (PTEC), immortalized proximal tubulus epithelial cells (IM-PTEC), RAW 264.7 macrophages and AML12 hepatocytes.
(TIF)
