# Supplementary figures and images for: TLR9 Mediates Remote Liver Injury following Severe Renal Ischemia Reperfusion
Source: PLoS One. 2015 Sep 11;10(9):e0137511. doi: 10.1371/journal.pone.0137511 (PMC4567139; doi:10.1371/journal.pone.0137511)

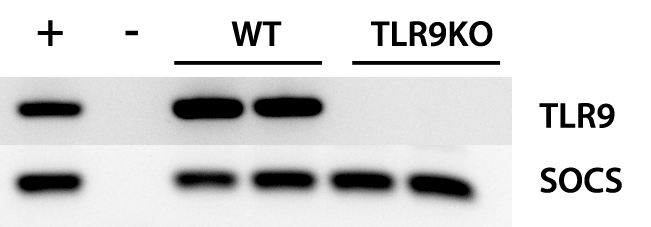

Supplement: S1 Fig — We randomly selected wild-type (Wt) and TLR9-deficient mice (TLR9KO) and analysed genomic DNA for the presence of the deletion in TLR9. SOCS was used as an endogenous reference. (TIF) [file pone.0137511.s001.tif]

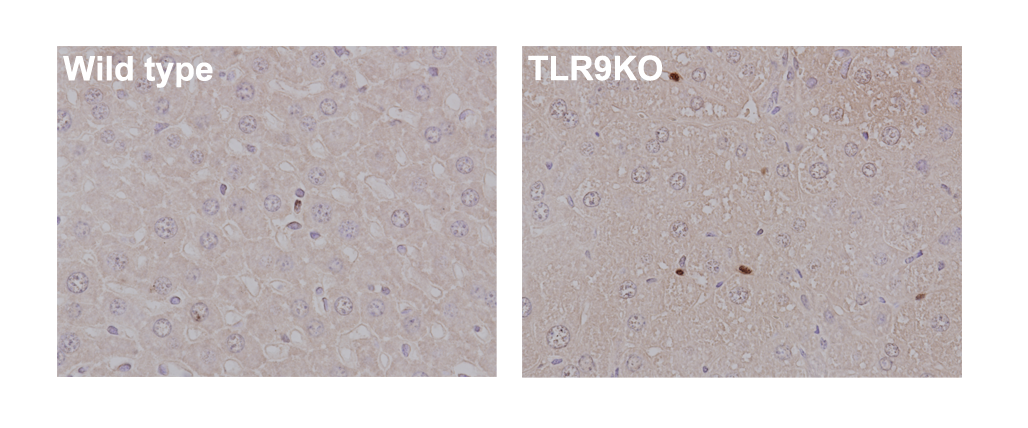

Supplement: S2 Fig — Liver sections of wild-type (Wt) and TLR9 deficient (TLR9KO) mice subjected to severe (30 minutes) ischemia and one day of reperfusion were analysed for Ly6G+ granulocyte accumulation at 400x magnification (high power field). (TIF) [file pone.0137511.s002.tif]
